# Detection of circulating tumor cells by means of machine learning using Smart-Seq2 sequencing

**DOI:** 10.1038/s41598-024-61378-8

**Published:** 2024-05-14

**Authors:** Krzysztof Pastuszak, Michał Sieczczyński, Marta Dzięgielewska, Rafał Wolniak, Agata Drewnowska, Marcel Korpal, Laura Zembrzuska, Anna Supernat, Anna J. Żaczek

**Affiliations:** 1 Faculty of Electronics, Telecommunication and Informatics, Gdańsk University of Technology, Gabriela Narutowicza 11/12, 80-233 Gdańsk, Poland; 2 Laboratory of Translational Oncology, Intercollegiate Faculty of Biotechnology, Medical University of Gdańsk, Marii Skłodowskiej-Curie 3a, 80-210 Gdańsk, Poland; 3 Centre of Biostatistics and Bioinformatics, Medical University of Gdańsk, Marii Skłodowskiej-Curie 3a, 80-210 Gdańsk, Poland

**Keywords:** Circulating tumor cells, CTC, Metastatic cancer, Single-cell sequencing, scRNA-seq, Machine learning, Artificial intelligence, Cancer, Computational biology and bioinformatics, Molecular biology, Molecular medicine, Oncology, Mathematics and computing

## Abstract

Circulating tumor cells (CTCs) are tumor cells that separate from the solid tumor and enter the bloodstream, which can cause metastasis. Detection and enumeration of CTCs show promising potential as a predictor for prognosis in cancer patients. Furthermore, single-cells sequencing is a technique that provides genetic information from individual cells and allows to classify them precisely and reliably. Sequencing data typically comprises thousands of gene expression reads per cell, which artificial intelligence algorithms can accurately analyze. This work presents machine-learning-based classifiers that differentiate CTCs from peripheral blood mononuclear cells (PBMCs) based on single cell RNA sequencing data. We developed four tree-based models and we trained and tested them on a dataset consisting of Smart-Seq2 sequenced data from primary tumor sections of breast cancer patients and PBMCs and on a public dataset with manually annotated CTC expression profiles from 34 metastatic breast patients, including triple-negative breast cancer. Our best models achieved about 95% balanced accuracy on the CTC test set on per cell basis, correctly detecting 133 out of 138 CTCs and CTC-PBMC clusters. Considering the non-invasive character of the liquid biopsy examination and our accurate results, we can conclude that our work has potential application value.

## Introduction

Liquid biopsies are non-invasive biopsies in which the material comes from a liquid sample (typically blood) collected from a donor. They represent an emerging area of research dedicated to cancer detection, prognosis prediction and therapy personalization. Circulating tumor cells (CTCs) are cells that have separated from the tumor and entered the bloodstream. CTC enumeration is one of the first FDA-approved methods based on the liquid biopsies used for stratifying cancer patients according to their prognosis. Common approaches are based on image analysis. Circulating tumor cells constitute a very small subset of cells from the primary tumor and are very scarce within the bloodstream. Further research into the biology of CTCs is warranted since they remain one of the important mechanisms of metastasis formation and their profiles have been shown to differ from those of cells in the primary tumor. Fully isolating CTCs from a mixture containing PBMCs remains challenging, as conventional approaches often damage the cells or potentially affect their transcriptomic landscape. Hence, CTCs should remain intact in order to obtain accurate and reliable data on their transcriptomics. Simultaneously, data labeling required to train a machine learning classifier poses a challenge. In general, several methods are known in the literature to detect CTCs; however, most are image-based and focus on cell enumeration rather than isolation for further transcriptomic profiling. Classification based on the transcriptomic profiles would resolve this problem, as the only data missing would be the correct label, provided by the classifier itself. Classification driven by single markers specific to CTCs, such as EpCAM, is characterized by less than ideal sensitivity^1,2^. Most of the markers commonly used in detection of CTCs are epithelial in nature, while a subset of CTCs demonstrate more mesenchymal properties^3^. Furthermore, protein expression does not always correlate strongly with mRNA expression. We evaluate the feasibility of machine learning based approaches towards CTC classification based on their transcriptomic profiles from single-cell RNA sequencing (scRNA-seq) data.

Gene expression data extracted during single-cell sequencing provides useful information about biological processes in a cell, enabling cell type identification and further analysis of their transcriptomic profiles without using aggressive isolation protocols, which could damage the cells affecting the results of further analysis. In contrast to a custom cytosensor-based approach, once extracted, data can be reused in many genetic analyses and this method only requires equipment that is already present in many medical centers.

During the literature review, we found multiple studies on CTC detection using artificial intelligence methods (not only based on scRNA-seq data)^4–9^. However, we observed several concerns regarding the reliability of the results presented in these studies. In particular, certain studies conducted testing with data derived from the same patients used for model training, potentially leading to the classifiers learning patient-specific cancer cell features rather than generalizable traits^6,7^. Furthermore, some investigations employed notably small datasets, such as those containing only 67 cells’ gene expression profiles or relied on samples collected from an insufficient number of patients^8^. Moreover, many authors failed to provide crucial metrics of their models, hindering comparisons between methods described in the papers.

There was a high level of variability in balance between sensitivity and specificity in analyzed papers, which made the direct comparison of results harder. Furthermore, focusing mainly on accuracy scores in case of imbalanced datasets (CTC datasets are usually highly imbalanced) makes it harder to evaluate and compare the methods. For example, if PBMCs are 9 times more prevalent in the dataset than CTCs, then a model, which classifies all cells as PBMCs, reaches an accuracy of 90%. Relatively high accuracy values do not necessarily correspond with models that perform well^10^. Some studies on CTC detection addressed crucial aspects of the mentioned problems^8^, but utilized image-based approaches.

In our work, we addressed all of the aforementioned issues. Instead of an accuracy score, we used balanced accuracy. We also included metrics: ROC AUC, precision, recall and F1 score. We used a dataset containing CTCs, PBMCs and CTC-PBMC clusters collected from 34 metastatic breast cancer patients. We have compared the performance of our models against the EpCAM based classification, which is one of the standards in CTC detection. Additionally, due to the scarcity of publicly available scRNA-seq data from CTCs, we decided to explore the feasibility of training a machine learning model focused on CTC detection using an artificial dataset comprised of a mixture of cells from primary tumor biopsies and PBMCs. We prepared a dataset spanning 29 154 cells’ gene expression profiles, where 1534 cells were tumor cells, and the diseased to healthy cell count ratio corresponded to a possible realistic ratio following a less strict CTC isolation protocol. PBMC samples were collected from 6 patients. We tested the models against actual CTC and PBMC mixtures from metastatic breast cancer patients. In our work, we also compared many state-of-the-art machine learning algorithms and achieved promising results. We also analyzed different feature selection methods.

## Materials and methods

**Figure 1:**
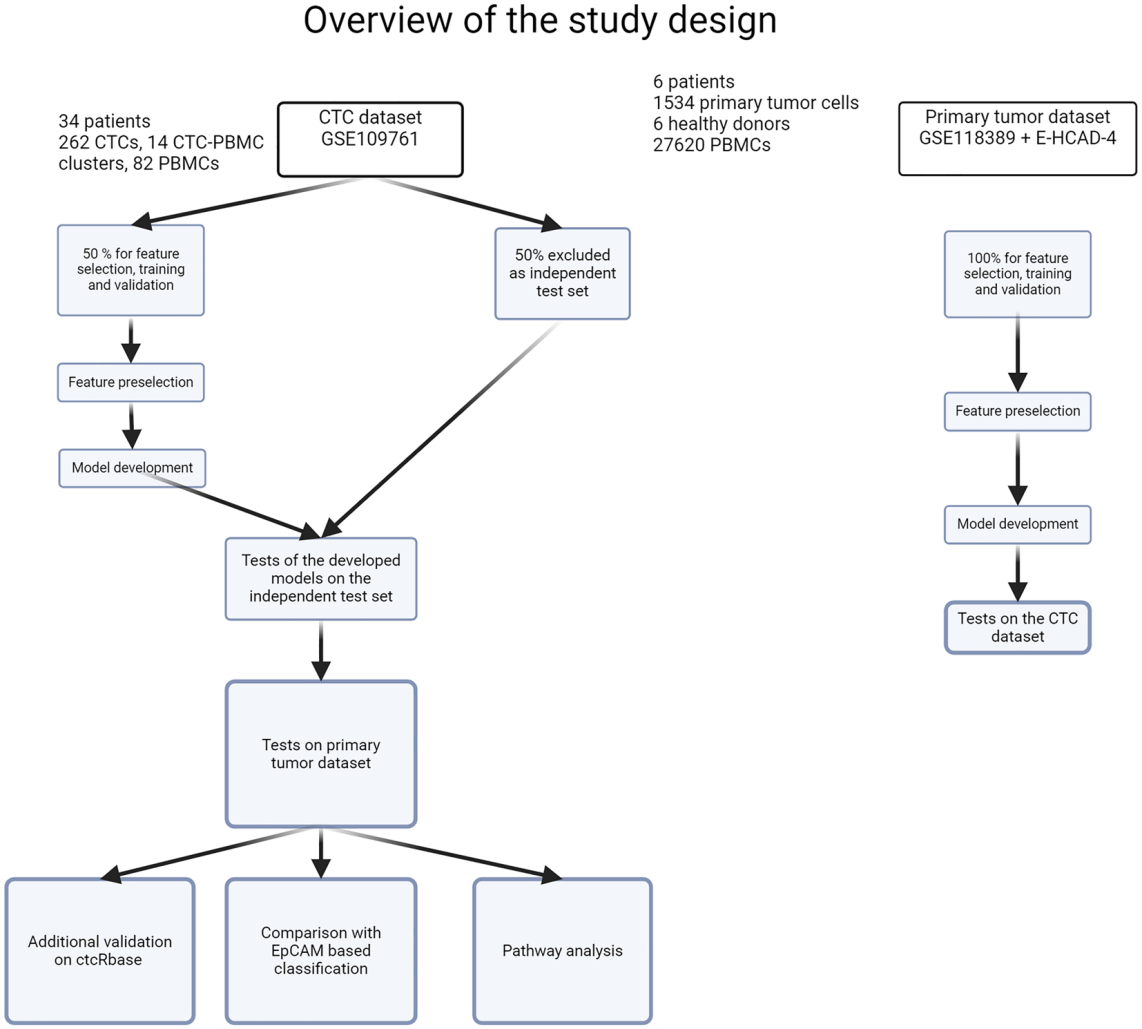
Overview of the study design.

An overview of the study design is presented in Fig. 1.

### Datasets

We selected three publicly available datasets for the study. The first dataset (deposited in GEO under accession number GSE109761) consisted of a mixture of 262 CTCs, 14 CTC-PBMC clusters and 82 PBMCs gathered from 34 metastatic breast cancer patients (including 5 triple-negative breast cancer patients, 16 ER+/PR+/HER2− patients and 1 ER−/PR−/HER2+ patient) sequenced using SmartSeq2^11^. Dataset GSE118389^10^ consisted of 1534 tumor cells gathered from 6 primary triple-negative breast cancer patients (TNBC) (one BRCA2 positive, four BRCA 1/2 negative, and one with unknown BRCA status). Cells were sequenced using SmartSeq2^12^. For the healthy cells, we selected 27,620 PBMCs from 6 healthy donors deposited in the Single Cell Expression Atlas (Census of Immune Cells experiment, id E-HCAD-4)^13^.

A primary tumor dataset was created using tumor cells from TNBC patients and PBMCs dataset, mimicking a mixture of CTCs and PBMCs. The constructed dataset featured a significant class imbalance, with a ratio of nearly 19:1 between healthy and diseased cells, which posed a challenge for the effective detection of outliers. This level of class disproportion is intended to represent the possible proportions between cell types in a less strict CTC isolation protocol, hence the chosen approach. To validate the results, algorithms were tested on a dataset containing single-cell expressions of CTCs and PBMCs from metastatic breast cancer patients. Visualization of the CTC dataset is presented in Supplementary Fig. 1.

### Data preparation

Single cell RNA-seq datasets were integrated using Seurat R package (version, 4.2.0)^14^. Each cell was characterized by the same set of features consisting of normalized expression levels. Only features present in every dataset were considered. Integrated data were then normalized and log-transformed. Features represented across a low percentage of the cells were removed from the dataset. The analysis only included cells with more than 200 and less than 2,500 distinct features expressed. Cells with more than 5% of read counts originating from mitochondrial material were excluded from the analysis. Samples from test sets underwent the same normalization process as the data from the training sets.

### Data splits

For the development of classifiers trained on actual CTC data, we randomly selected 50% of the CTC dataset, maintaining class proportions for training and validation purposes, while using the remaining cells as an independent test set. CTC-PBMC clusters were considered as CTCs. We employed a threefold cross-validation during the training process. The same splits were utilized for each combination of algorithm and feature preselection method. Consequently, our stratified validation set comprised 1/3 of the training set and varied with each cross-validation iteration. We further assessed the performance of the developed models using the primary tumor dataset. The details of dataset splits are presented in Table 1.

**Table 1:** Overview of the data splits used in the study.

	Training and validation	Test set I	Test set II
	CTC dataset	CTC dataset	Primary tumor dataset
Cancer cells	130 + 8 clusters	132 + 6 clusters	1534
Blood cells	38	43	27,620
Source	50% of GSE109761	50% of GSE109761	GSE118389 + E-HCAD-4

Additionally, we conducted experiments in which we used only the primary tumor dataset to train the models, with the entire CTC dataset serving as an independent test set.

### Feature selection

After the initial prefiltering of transcripts during the quality control and dataset integration process, the datasets remained highly dimensional, containing over 15,000 features. This substantial imbalance between the number of features and the number of samples may lead to reduced performance of the developed classifiers. Additionally, a significant percentage of transcript expression levels were predominantly close to zero. As a result, we conducted further dimensionality reduction on the datasets.

Our focus was on reducing the number of transcripts included in the analysis, rather than computing projections of original features on artificial ones. To prevent potential information leakage, only the training set was subjected to statistical analysis. We evaluated two approaches for feature selection. In the selection based on mean expression per class, we eliminated features with mean expression levels above the assumed threshold in each class. In the second approach, using two criteria for selection, we incorporated features characterized by mean expression above the cutoff in the entire dataset and high expression levels in at least one cell. The thresholds were determined experimentally using training and validation data.

Following the feature selection process, the datasets contained between 46 and 67 transcripts, depending on the dataset and filtering method. The exact feature numbers are presented in Supplementary Table 1. Supplementary Fig. 1 displays the t-SNE visualization of CTC training set after filtering, and additional visualizations are provided in Supplementary Figs. 2 and 3.

### Model reduction

After building and validating the models in the previous stages, we identified the top-performing ones for both scenarios (training on the primary tumor dataset and training on a subset of real CTC data). We then analyzed the feature importance of these models, including only those features that surpassed the predetermined threshold in the final feature sets. Subsequently, we trained new models on these reduced feature sets.

### Classification algorithms

The data under consideration is tabular. Our focus was on tree based and gradient boosting based classification algorithms, which tend to perform well on the tabular data^15^ and allow for relatively easy analysis of feature importance and enable a relatively straightforward analysis of feature importance. We included in the study four different machine learning algorithms: Extreme Gradient Boosting (XGBoost, version 1.6.2)^16^, Light Gradient Boosting Machine (LightGBM, version 3.3.3)^17^, Random Forest (as implemented in scikit-learn, version 1.3.1)^18^ and Balanced Random Forest (as implemented in imbalanced-learn, version 0.11.0)^19^.

## Results

### Experiments

We conducted a series of experiments on both types of training data, employing a threefold cross-validation method for each of the algorithms. Specifically, the training set was divided into three parts, and three models were trained such that the validation set contained one unique part for each model, while the remaining data was used for training. The final results were an average of the individual model results. Due to the small size of the CTC training set and the highly imbalanced data, the number of folds was limited to 3. A larger number of folds might have led to an insufficient number of samples for each class in individual folds, potentially negatively impacting the training process. To evaluate the models, we used well-established metrics: balanced accuracy, area under the ROC curve (AUC), recall, precision, and F1 score.

### Performance of ML models

**Figure 2:**
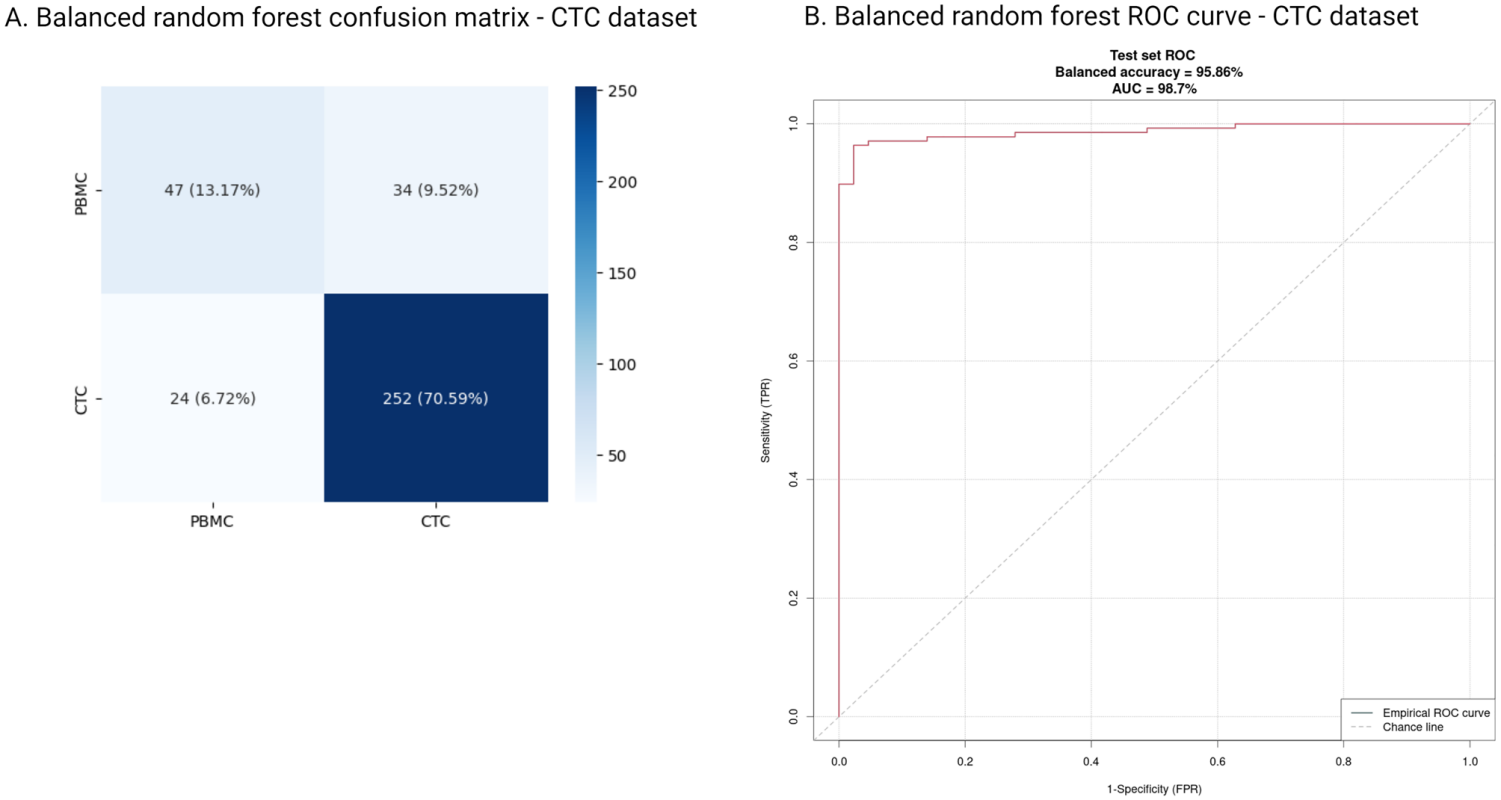
Performance of balanced random forest model trained on the CTC data as tested on the remaining part of the CTC dataset. (**A**) Confusion matrix. (**B**) ROC curve.

**Figure 3:**
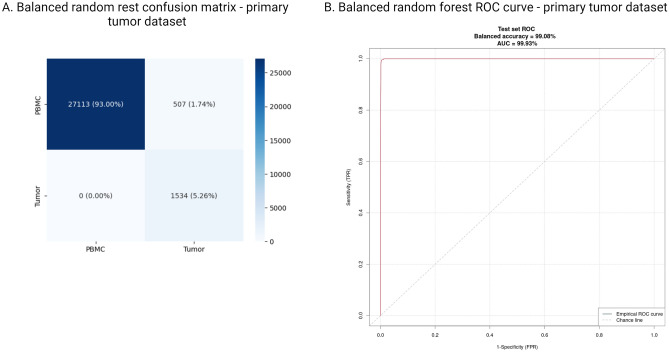
Performance of balanced random forest model trained on the real CTC data as tested on the primary tumor dataset. (**A**) Confusion matrix. (**B**) ROC curve.

In the first set of experiments, algorithms were trained using the CTC dataset, with half of the CTC data allocated for training and validation purposes. The performance was then assessed on both the remaining portion of the real CTC dataset and the primary tumor dataset (Table 2). In both test sets, the balanced random forest algorithm outperformed the other methods. The performance of the balanced random forest classifiers was notably high, achieving over 95% balanced accuracy on the CTC test set and 99% on the primary tumor set. The efficacy of both feature preselection methods was comparable when combined with the balanced random forest. Confusion matrices and ROC curves are displayed in Figs. 2 and 3 for the CTC test set and primary tumor test set, respectively. Boxplots illustrating the distribution of raw classification scores can be found in Supplementary Figs. 4 and 5. Interestingly, class separation was more evident in the real CTC data compared to the primary tumor dataset. Test set results for each combination of algorithm and feature preselection method are provided in Supplementary Table 2.

**Table 2:** Performance of balanced random forest with different feature preselection methods for the classifiers trained and validated on the subset of CTC data.

	Two criteria selection				
	Balanced accuracy	ROC AUC	Precision	Recall	F1 score
CTC test set	0.96	0.99	0.99	0.96	0.97
Primary tumor test set	0.99	0.99	0.99	0.99	0.99

	Selection based on mean expression per class				
	Balanced accuracy	ROC AUC	Precision	Recall	F1 score
CTC test set	0.95	0.99	0.98	0.96	0.97
Primary tumor test set	0.99	0.99	0.99	0.99	0.99

The classifiers were trained using half of the real CTC data.

### Feature importance analysis

In order to further enhance and validate the model, we examined the feature importance of variables incorporated in the constructed model. We chose 22 features with an importance level greater than 0.01 and used them to train a reduced-size model. The list of included features along with their importance levels can be found in Supplementary Table 4. The detailed performance of the final model is displayed in Table 3. Despite the reduction in the number of features, the final model achieved a performance similar to the original one. Confusion matrices, ROC curves, and boxplots illustrating the distribution of raw classification scores for the final model are shown in Supplementary Figs. 6 and 7 for the CTC test set and primary tumor test set, respectively.

**Table 3:** Performance of model trained using the reduced feature sets.

	Balanced random forest trained on CTC data				
	Balanced accuracy	ROC AUC	Precision	Recall	F1 score
CTC test set	0.96	0.98	0.99	0.97	0.98
Primary tumor test set	0.99	1	0.88	0.99	0.94

### EpCAM based classification

To confirm the utility of machine learning-based models in detecting CTCs, their performance was compared with EpCAM-based classification. EpCAM is a marker commonly used in detection of CTCs^1^. The threshold for decision-making was set at the level of EpCAM characterized by the maximum value of the Youden's index. EpCAM driven classification managed to reach 91% AUC with 89% balanced accuracy on the CTC test set and only 53.7% AUC on primary tumor set, as opposed to 95% balanced accuracy on the CTC test set and 99% on the primary tumor set. Detailed results are presented in Supplementary Table 5.

### External validation

To further validate the obtained results, additional tests were conducted with CTC and PBMC samples from public datasets aggregated in ctcRbase^20^. It should be noted that some of the constituent datasets were prepared using different laboratory techniques than the ones used in CTC dataset in this study. Models based on features from two criteria selection outperformed models based on features from mean expression per class selection. Despite the potential bias, each model trained with features from two criteria selection exceeded 94% AUC and 92% balanced accuracy, with the best models reaching 99% AUC with 97% specificity and 96% sensitivity. Detailed results are presented in Supplementary Table 6. ROC curves are depicted in Supplementary Fig. 8.

### Enrichment analysis

**Figure 4:**
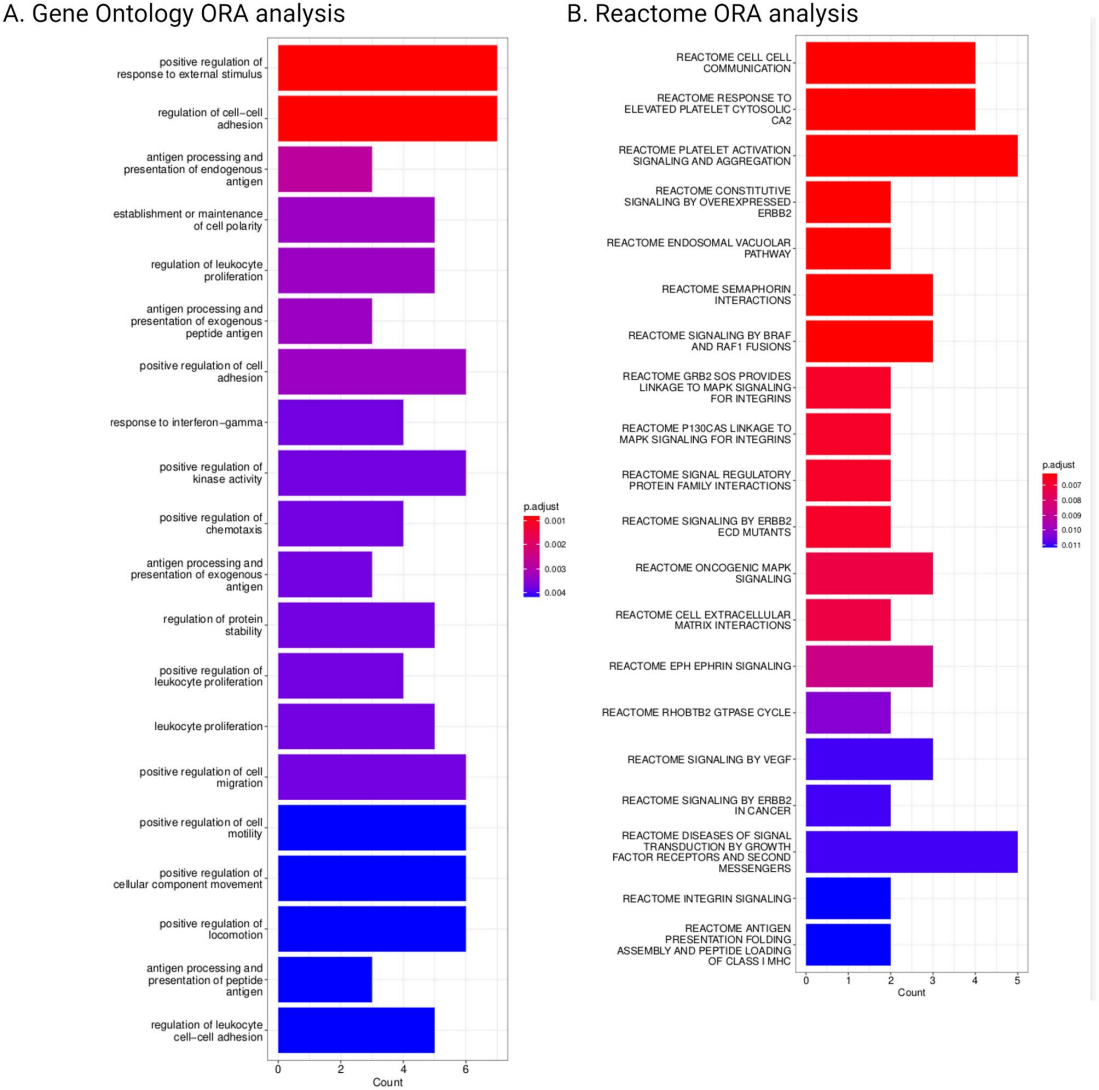
Enrichment analysis for the final feature set. (**A**) Gene ontology. (**B**) Reactome.

We performed Gene Ontology (GO)^21^ and Reactome^22^ pathway enrichment analysis for the set of final features. The results are presented in Fig. 4. The most enriched GO pathways were related to cell adhesion or immune function. Reactome analysis also revealed enrichment related to cell signaling processes, platelet activation and cell-to-cell communication. The observed patterns are consistent with the literature dedicated to CTCs. Interactions between CTCs and immune system, either in form of direct cell-to-cell interaction, or indirectly through released molecules, have been shown to be essential for CTC survival in blood^23^. The adhesion of platelets to CTCs is considered one of the significant mechanisms enabling CTCs to evade recognition and destruction by NK cells^24^. Most aggressive CTCs capable of metastasis induction seem to be characterized by high cell plasticity^3^. Both epithelial-to-mesenchymal transition (EMT) and mesenchymal-to-epithelial transition (MET) are observed in CTCs^3,25^.

### Training model on primary tumor dataset

We conducted an additional set of experiments where we utilized the primary tumor dataset for training and validation purposes to investigate the feasibility of training CTC detection algorithms on data coming from tumor biopsies. The entire CTC dataset was employed as the independent test set.

**Figure 5:**
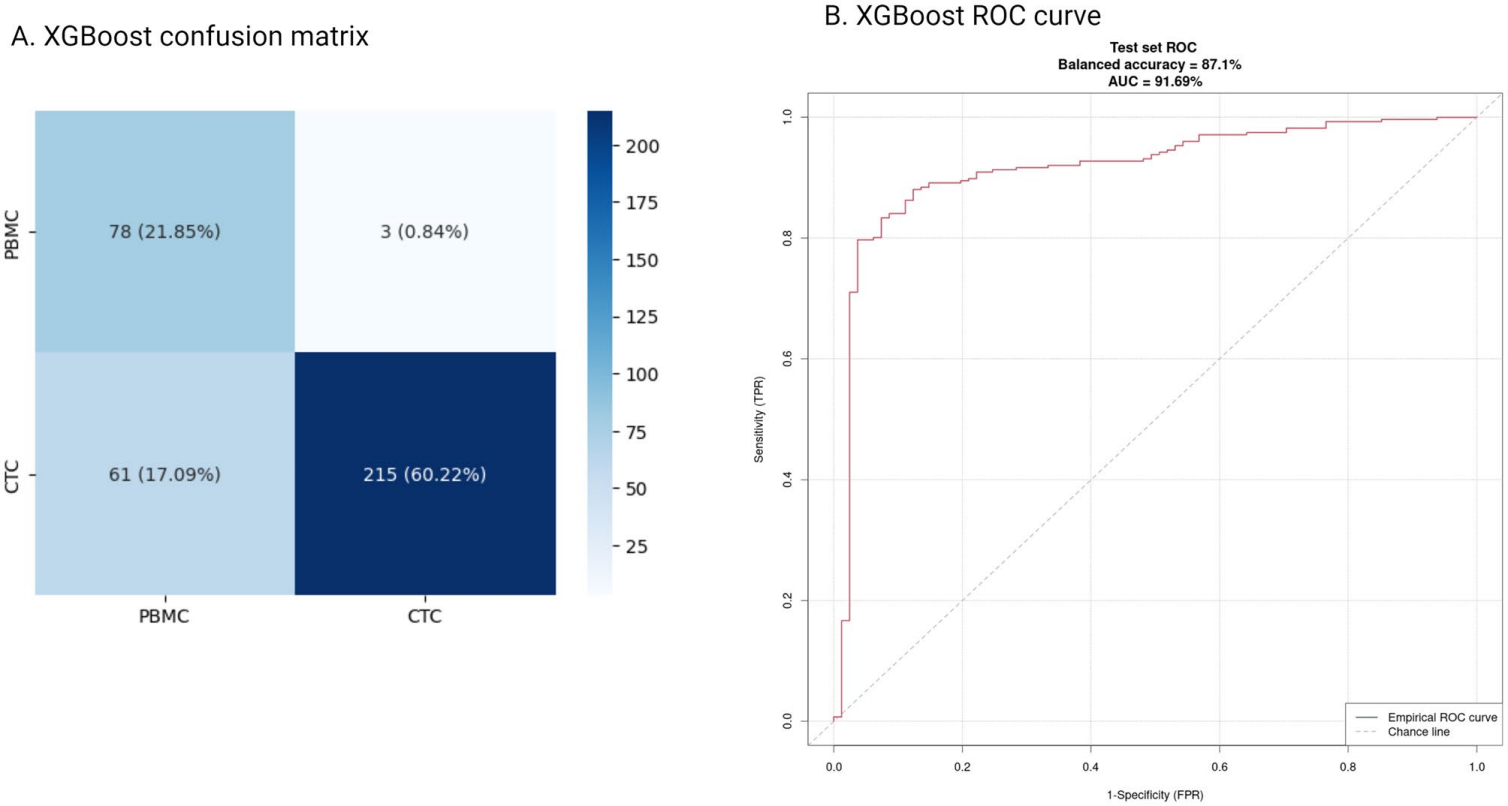
Performance of XGBoost model trained on the primary tumor dataset as tested on the whole CTC dataset. (**A**) Confusion matrix. (**B**) ROC curve.

Gradient-based models demonstrated the best performance, with XGBoost slightly surpassing LightGBM (91% AUC vs 89% AUC). Detailed performance metrics are presented in Table 4. The confusion matrix and ROC curve for the test set, representing the best combination of methods, are shown in Fig. 5. Boxplots depicting distribution of raw classification scores among classes are presented in Supplementary Fig. 9. Results on the independent test set (whole CTC dataset) for each combination of algorithm and feature preselection method are presented in Supplementary Table 3.

**Table 4:** Performance of models trained on primary tumor dataset, as tested on the entire CTC dataset.

Classifier/metric	Two criteria selection				
	Balanced accuracy	ROC AUC	Precision	Recall	F1 score
XGBoost	0.72	0.85	0.96	0.52	0.67
LightGBM	0.83	0.9	0.97	0.74	0.84

Classifier/metric	Selection based on mean expression per class				
	Balanced accuracy	ROC AUC	Precision	Recall	F1 score
XGBoost	**0.85**	**0.91**	**0.98**	**0.76**	**0.85**
LightGBM	0.83	0.89	0.96	0.76	0.85

Performance of the best performing model is in bold.

## Discussion

There are only a few studies on a machine learning based classification of CTC gene expression profiles^4,8,26,27^. One study achieved a mean accuracy of approximately 93% for CTC versus PBMC classification using the Gradient Boosting Machines algorithm^8^, which is notably lower than the results obtained with our best approach. Another study reported an accuracy of 81.9% for CTC classification; however, this was based on gene expression microarray data. In a related study using similar microarray data, accuracies of 95.16% and 96.05% were attained for classifying healthy control groups versus non-metastatic patient groups^26^ and control groups versus metastatic cancer groups^26^, respectively. Nevertheless, the data in these studies were derived from cancer tissue rather than CTCs.

A superior score was obtained by incorporating additional knowledge from biological networks^4^, where an average AUC score of 99.95% was achieved using the SVC-RBF (Support Vector Classifier using Radial Basis Function as a kernel function) algorithm for CTC versus PBMC classification. However, the dataset employed in this study was quite limited, containing only eight PBMC profiles and five CTC profiles, which likely significantly impacted the score. Despite this, the utilization of a biological network encoding gene proximities^4^ presents an interesting concept that could potentially enhance the classification metrics based on gene expression data in future research.

Our top-performing model outperformed many machine learning models that employed various types of data ^6,9,28^. A neural network trained on fluorescence microscopy images achieved a sensitivity of 92.6% and specificity of 90.8% in CTC identification ^6^, while a convolutional neural network utilized for CTC detection on microscopy images obtained a sensitivity of 90.3% and a specificity of 91.3% ^9^. In contrast, another study reported lower results, with 63% sensitivity and 68.4% specificity, using immunofluorescence images of CTCs and artificial intelligence methods^19^. Furthermore, research has been conducted on CTC detection using Raman spectroscopy^29–31^. The most effective CTC detection models identified in our review achieved a sensitivity of 97.8% and specificity of 100% on serum biofluid data collected from prostate cancer patients^29,30^ as well as a sensitivity of 94.4% and specificity of 100% on plasma biofluid data from esophageal cancer patients^29,31^. However, the similarity of cancer cells might have influenced the classification scores in these cases. Both studies employed Support Vector Machine (SVM) models^29–31^. Additionally, SVM classification from impedance cytometer data attained a sensitivity of 96.6% and specificity of 99.8% in the classification of white blood cells (WBCs) versus breast tumor cells from the MCF7 cell line^7^.

The AdnaTest is a commonly used CE-IVD system for CTC isolation and detection^32,33^. However, it examines only a limited number of tumor-specific marker genes^33^, including one gene, ERBB2, which is common to our panel. The AdnaTest has a detection frequency of 62%^33^, which is comparable to other available methods^32,33^ but yields lower scores than our approach. Machine learning classification of gene expression profiles allows for predictions based on a substantially larger number of biomarkers, where each gene possesses a probabilistic correlation with a cell type. Moreover, artificial intelligence (AI) techniques are proficient in identifying more complex relationships between genes and the target attribute. Consequently, AI algorithms employed in gene-based predictions hold significant potential to substantially enhance cancer diagnosis, including methods currently available on the market.

Models trained on actual CTC data demonstrated superior performance compared to those trained on artificial datasets. This discrepancy may be attributed to multiple factors. CTCs are cells that have undergone a selection process based on their molecular properties and might not represent typical primary tumor cells. Furthermore, primary tumor sections were acquired from triple-negative breast cancer (TNBC) patients, while CTCs were obtained from patients with a mix of molecular breast cancer subtypes, which constitutes a limitation of the study. The sections could also comprise various healthy cells, such as fibroblasts or normal breast tissue cells, which likely impact the results. Based on our findings, we conclude that it is more beneficial to use real CTC data to train classification models in future studies. Another limitation of the study is the small number of CTC datasets utilized, primarily due to the scarcity of publicly available data.

We further validated the final model by performing enrichment analysis. Features included in the model were related to the immune function, which is expected in case of PBMCs, signaling pathways, which are often highly abnormal in cancer cells^34^ and cell-to-cell adhesion.

It should be noted that machine learning methods often exhibit sensitivity to the presence of batch effects. Caution should be prioritized when applying developed models to data stemming from experiments where the laboratory protocols significantly diverge from those employed in the datasets under consideration within this study. Validation experiments on public datasets, varying in laboratory methodologies used, have demonstrated the robustness of the developed models' performance.

Nevertheless, prior to applying the developed algorithms to cells sequenced via alternative techniques, it is advised to conduct additional validation of the models using datasets prepared with the aforementioned laboratory methodologies. This precaution becomes particularly important when the employed method results in significantly different distributions of acquired reads, as exemplified in the case of Chromium 10x platform, where cells typically exhibit considerably lower read counts compared to the SmartSeq method.

## Conclusions

In this article, we presented accurate methods for CTC detection using machine learning algorithms based on single-cell sequencing data. Specifically, we analyzed two feature selection methods and four classification algorithms. Our best-performing solution was tested using a dataset containing CTC and PBMC expression profiles from a cohort of 34 metastatic breast cancer patients and a primary tumor dataset constructed by merging PBMC datasets with tumor sections from TNBC patients. The algorithm achieved a balanced accuracy close to 96% on the real CTC data. Our models outperformed EpCAM based classification and their performance was confirmed on external datasets. Our best results were obtained for models with a significantly reduced number of features, which not only increased the accuracy of our solution but also its speed. Considering the advantages of our results, we conclude that it has potential application value.

## Supplementary Information

Supplementary Tables.

Supplementary Legends.

Supplementary Figure 1.

Supplementary Figure 2.

Supplementary Figure 3.

Supplementary Figure 4.

Supplementary Figure 5.

Supplementary Figure 6.

Supplementary Figure 7.

Supplementary Figure 8.

Supplementary Figure 9.

## Data Availability

The data that was used in the study are openly available in GEO under accession number GSE109761, at https://www.ncbi.nlm.nih.gov/geo/query/acc.cgi?acc=GSE109761.

## Code availability

Code available at https://github.com/melehean/CTC-Detection.
