# Supplementary material for: Detection of circulating tumor cells by means of machine learning using Smart-Seq2 sequencing
Source: Sci Rep. 2024 May 14;14:11057. doi: 10.1038/s41598-024-61378-8 (PMC11094170; doi:10.1038/s41598-024-61378-8)
Supplement: Supplementary file 1 — Supplementary Tables. [file 41598_2024_61378_MOESM1_ESM.docx]

Accurate detection of circulating tumor cells by means of machine learning

**Supplementary Tables**

**Supplementary Material, Table S1** Number of features selected based on training sets depending on filtering method.

|  | **Two criteria selection** | **Selection based on mean expression per class** |
| --- | --- | --- |
| **Primary tumor training set** | 46 | 59 |
| **CTC training set** | 67 | 67 |

**Supplementary Material, Table S2** Performance of different classification algorithms and feature preselection methods for the classifiers trained and validated on the subset of CTC data. The classifiers were trained using half of the real CTC data. Results are presented for the independent test set which consisted of the remaining part of CTC data.

|  | **Two criteria selection** | | | | |
| --- | --- | --- | --- | --- | --- |
| **Classifier/metric** | **Balanced accuracy** | **ROC AUC** | **Precision** | **Recall** | **F1 score** |
| XGBoost | 0,78 | 0,99 | 0,42 | 0,71 | 0,31 |
| LightGBM | 0,88 | 0,98 | 0,27 | 0,99 | 0,4 |
| Random Forest | 0,79 | 1 | 0,2 | 1 | 0,3 |
| **Balanced Random Forest** | **0,99** | **0,99** | **0,9** | **0,99** | **0,94** |
|  | **Selection based on mean expression per class** | | | | |
| **Classifier/metric** | **Balanced accuracy** | **ROC AUC** | **Precision** | **Recall** | **F1 score** |
| XGBoost | 0,94 | 0,99 | 0,4 | 1 | 0,54 |
| LightGBM | 0,9 | 1 | 0,26 | 1 | 0,4 |
| Random Forest | 0,81 | 0,99 | 0,13 | 1 | 0,23 |
| **Balanced Random Forest** | **0,99** | **1** | **0,9** | **0,99** | **0,94** |

**Supplementary Material, Table S3** Performance of different classification algorithms and feature preselection methods for the classifiers trained and validated on the primary tumor dataset/ Results are presented for the independent test set which consisted of the entire CTC dataset.

|  | **Two criteria selection** | | | | |
| --- | --- | --- | --- | --- | --- |
| **Classifier/metric** | **Balanced accuracy** | **ROC AUC** | **Precision** | **Recall** | **F1 score** |
| XGBoost | 0,72 | 0,85 | 0,96 | 0,52 | 0,67 |
| LightGBM | 0,83 | 0,9 | 0,97 | 0,74 | 0,84 |
| Random Forest | 0,62 | 0,84 | 0,97 | 0,27 | 0,42 |
| Balanced Random Forest | 0,68 | 0,81 | 0,93 | 0,49 | 0,64 |
|  | **Selection based on mean expression per class** | | | | |
| **Classifier/metric** | **Balanced accuracy** | ROC AUC | Precision | Recall | F1 score |
| **XGBoost** | **0,85** | **0,91** | **0,98** | **0,76** | **0,85** |
| LightGBM | 0,83 | 0,89 | 0,96 | 0,76 | 0,85 |
| Random Forest | 0,81 | 0,89 | 0,98 | 0,68 | 0,8 |
| Balanced Random Forest | 0,75 | 0,9 | 0,89 | 0,91 | 0,9 |

**Supplementary Material, Table S4** List of features used in final, reduced size, balanced random forest model.

| Transcript | Importance |
| --- | --- |
| KRT19 | 0,1214 |
| HLA-E | 0,0789 |
| LMNA | 0,0672 |
| ACTG1 | 0,0546 |
| FTL | 0,0534 |
| PTK2 | 0,0516 |
| CD74 | 0,0511 |
| SPINT2 | 0,0472 |
| PTPN14 | 0,0445 |
| EFNA1 | 0,0361 |
| TMSB4X | 0,0317 |
| CD24 | 0,0292 |
| B2M | 0,0217 |
| ERBB2 | 0,0211 |
| SRGN | 0,0165 |
| CCL4 | 0,0149 |
| TLN1 | 0,0131 |
| SH3BGRL3 | 0,0129 |
| FLNA | 0,0127 |
| GAPDH | 0,0123 |
| SKAP2 | 0,0114 |
| HSP90AA1 | 0,0114 |

**Supplementary Material, Table S5** Results of EpCAM driven classification on the CTC dataset and the datasets included in the ctcRbase

| **Dataset** | **AUC** | **Balanced accuracy** | **Specificity** | **Sensitivity** | **Precision** |
| --- | --- | --- | --- | --- | --- |
| **CTC dataset** | 0.91 | 0.89 | 0.99 | 0.80 | 0.59 |
| **ctcRbase** | 0.66 | 0.69 | 0.95 | 0.44 | 0.25 |

**Supplementary Material, Table S6** Performance of models trained on CTC as tested on datasets included in the ctcRbase

| **Algorithm** | **AUC** | **Accuracy** | **Balanced accuracy** | **Specificity** | **Sensitivity** | **Precision** |
| --- | --- | --- | --- | --- | --- | --- |
| **Balanced random forest** | 0.94 | 0.89 | 0.92 | 0.96 | 0.87 | 0.60 |
| **XGBoost** | 0.97 | 0.92 | 0.94 | 0.97 | 0.91 | 0.68 |
| **Random forest** | 0.99 | 0.98 | 0.96 | 0.93 | 0.99 | 0.97 |
| **LightGBM** | 0.99 | 0.96 | 0.96 | 0.97 | 0.96 | 0.81 |
