# Supplementary material for: Detection of circulating tumor cells by means of machine learning using Smart-Seq2 sequencing
Source: Sci Rep. 2024 May 14;14:11057. doi: 10.1038/s41598-024-61378-8 (PMC11094170; doi:10.1038/s41598-024-61378-8)
Supplement: Supplementary file 2 — Supplementary Legends. [file 41598_2024_61378_MOESM2_ESM.docx]

**Supplementary Material**, **Figure S 1**. TSNE projection of CTC dataset following feature filtering by mean and maximum expression level.

**Supplementary Material**, **Figure S2** TSNE projection of both datasets following feature filtering by two criteria selection. (A) primary tumor dataset. (B) CTC dataset.

**Supplementary Material**, **Figure S3** TSNE projection of both dataset following feature filtering by mean per class expression level. (A) primary tumor dataset. (B) CTC dataset

**Supplementary Material**, **Figure S4** Performance of balanced random forest model trained on the CTC data as tested on the remaining part of the CTC dataset - distribution of raw classification scores among classes.

**Supplementary Material**, **Figure S5** Performance of balanced random forest model trained on the CTC data as tested on the primary tumor dataset - distribution of classification scores among classes.

**Supplementary Material**, **Figure S6** Performance of the final balanced random forest model trained on the real CTC data as tested on the remaining part of the CTC dataset. (A) confusion matrix. (B) ROC curve.(C) distribution of classification scores among classes

**Supplementary Material**, **Figure S7** Performance of balanced random forest model trained on the CTC data as tested on the tumor biopsy dataset. (A) confusion matrix. (B) ROC curve.(C) distribution of classification scores among classes

**Supplementary Material**, **Figure S8** ROC curves of balanced random forest (A) and LightGBM (B) models as tested on the datasets included in ctcRbase

**Supplementary Material**, **Figure S9** Performance of balanced random forest model trained on the primary tumor data as tested on the CTC dataset - distribution of raw classification scores among classes.
