# Supplementary figures and images for: Detection of circulating tumor cells by means of machine learning using Smart-Seq2 sequencing
Source: Sci Rep. 2024 May 14;14:11057. doi: 10.1038/s41598-024-61378-8 (PMC11094170; doi:10.1038/s41598-024-61378-8)

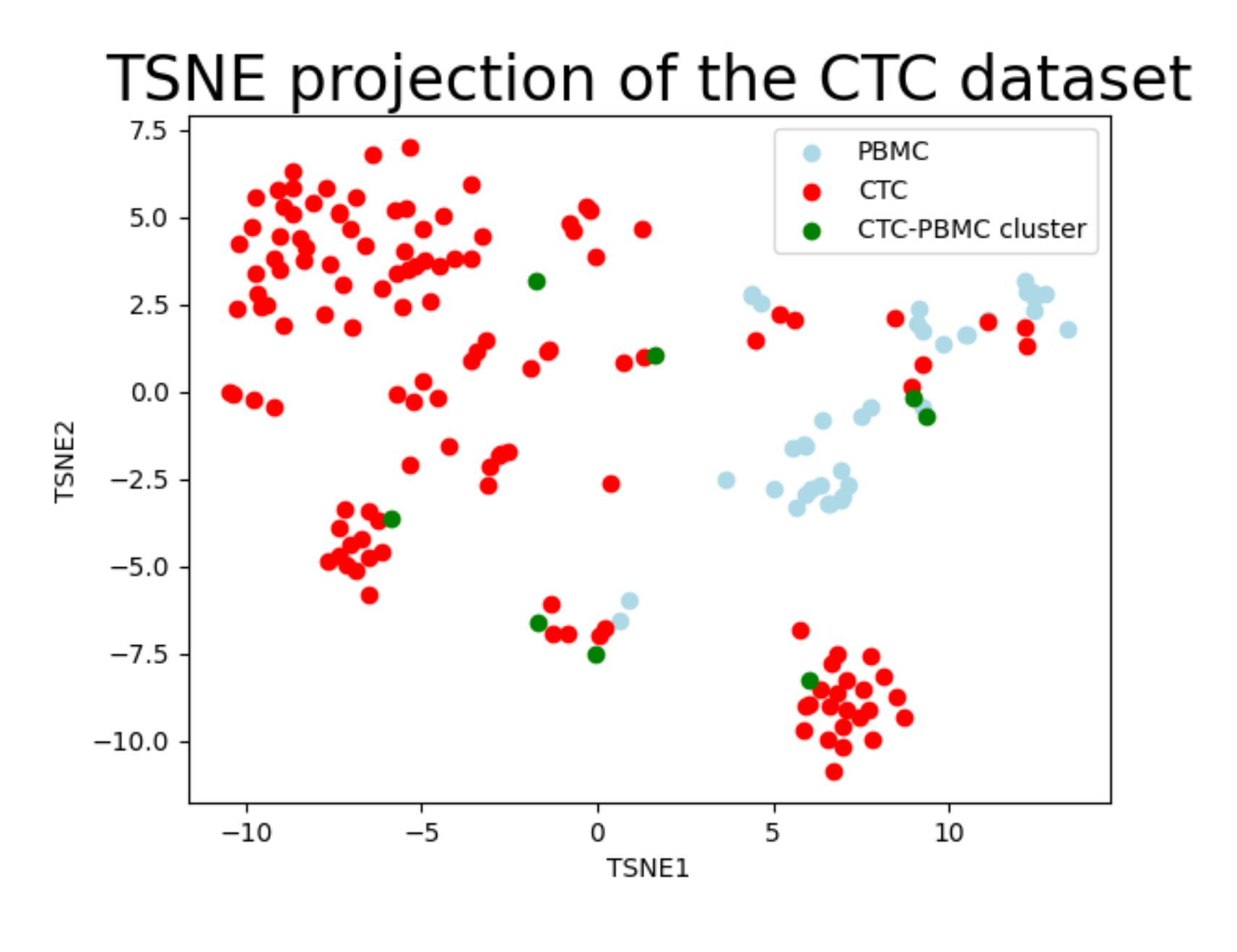

Supplement: Supplementary file 3 — Supplementary Figure 1. [file 41598_2024_61378_MOESM3_ESM.tiff]

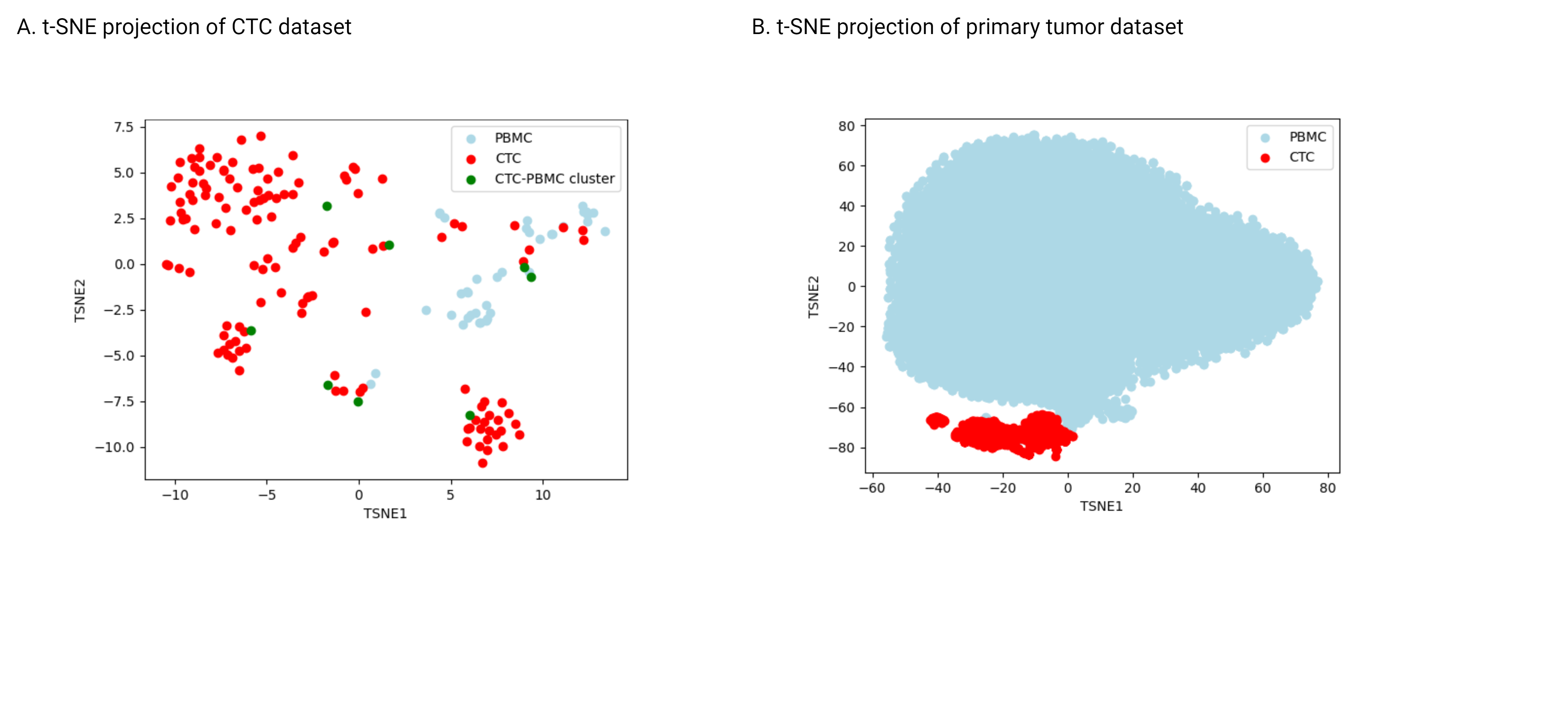

Supplement: Supplementary file 4 — Supplementary Figure 2. [file 41598_2024_61378_MOESM4_ESM.tiff]

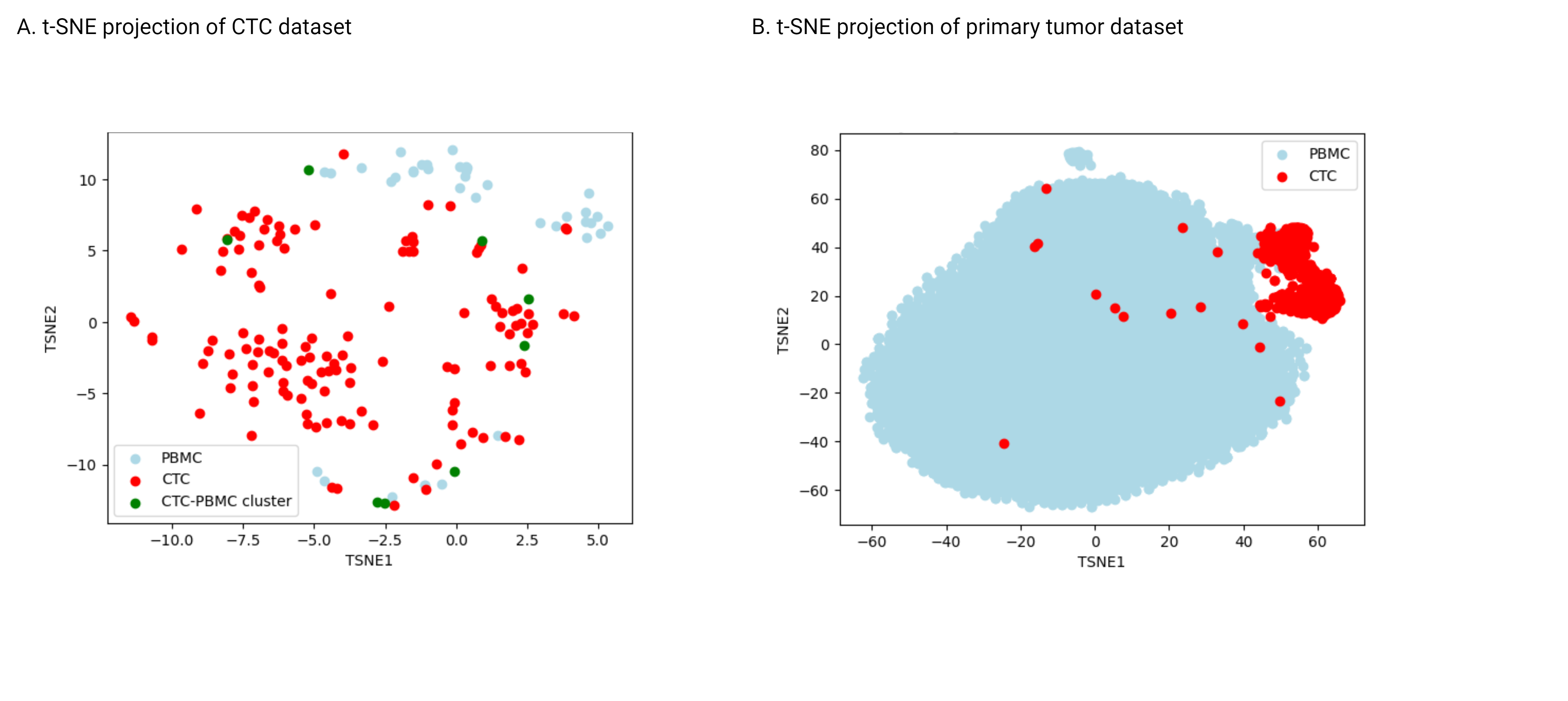

Supplement: Supplementary file 5 — Supplementary Figure 3. [file 41598_2024_61378_MOESM5_ESM.tiff]

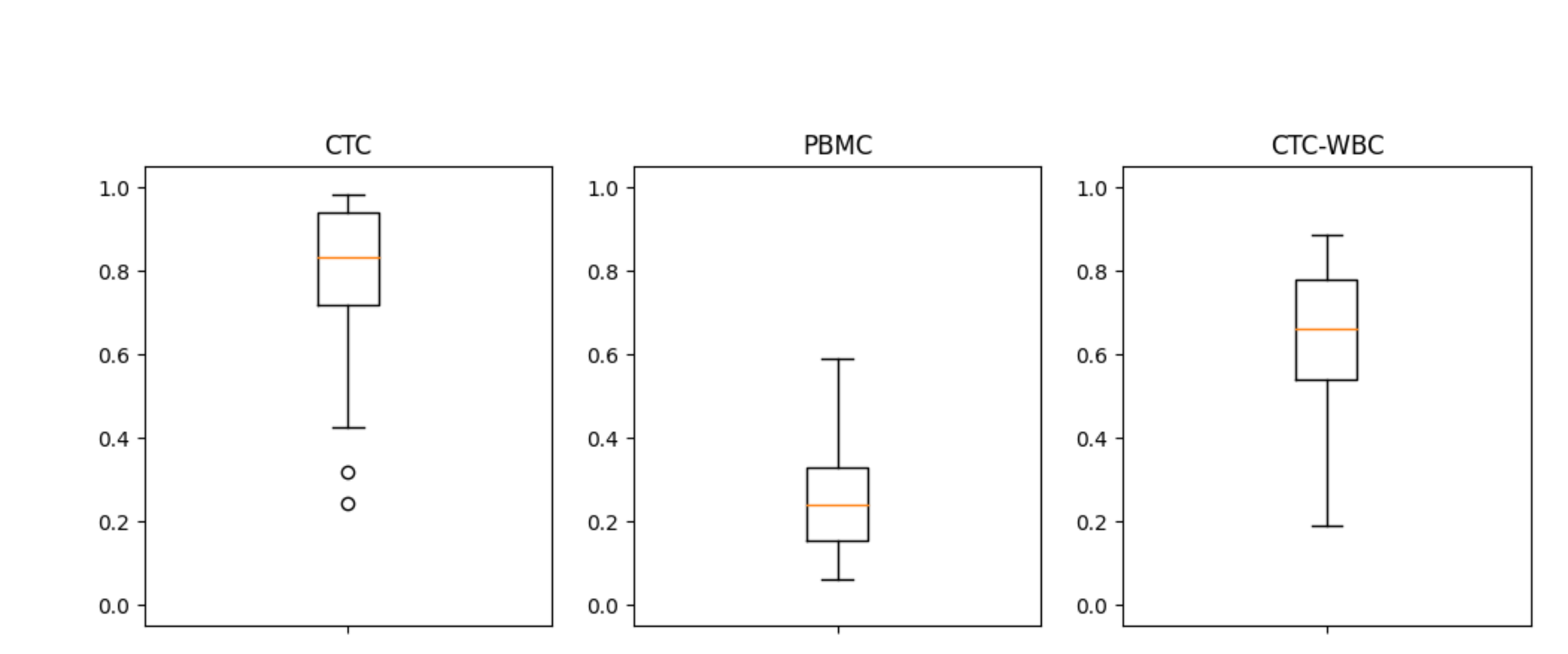

Supplement: Supplementary file 6 — Supplementary Figure 4. [file 41598_2024_61378_MOESM6_ESM.tiff]

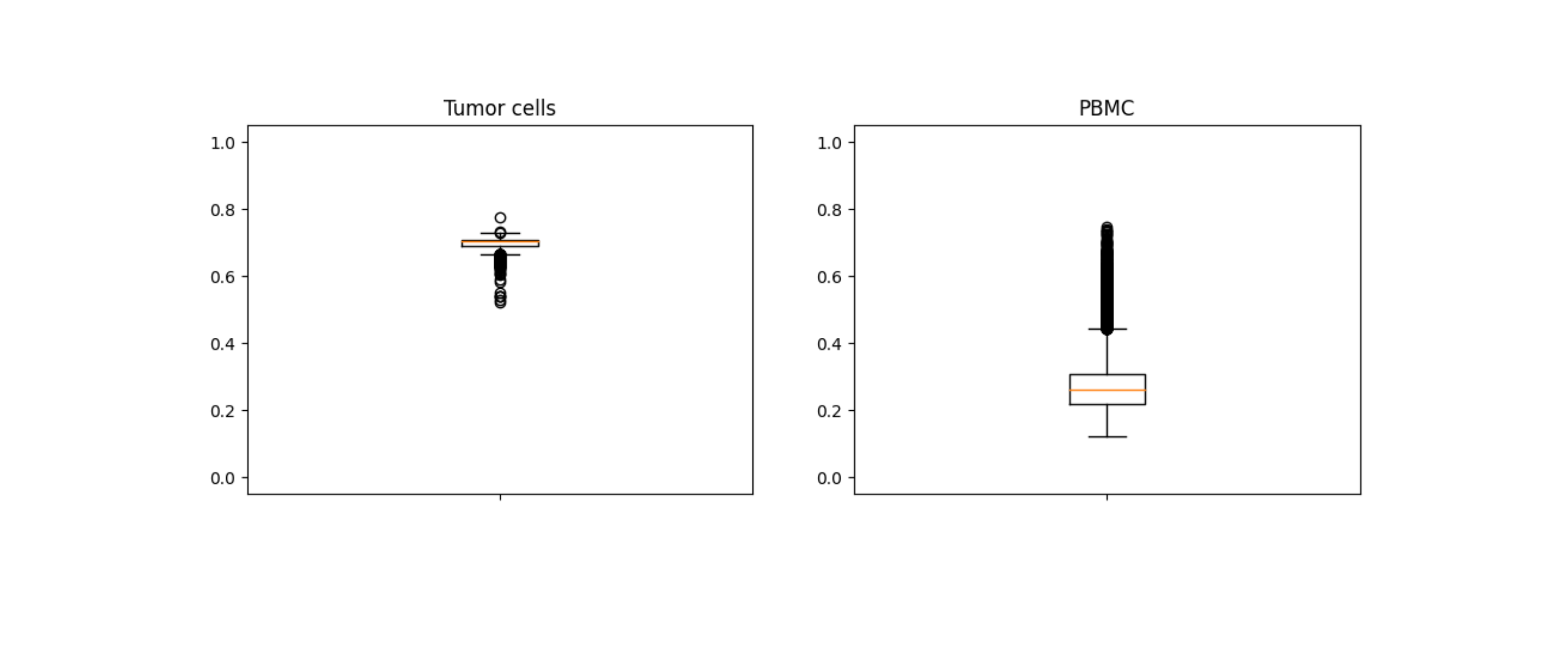

Supplement: Supplementary file 7 — Supplementary Figure 5. [file 41598_2024_61378_MOESM7_ESM.tiff]

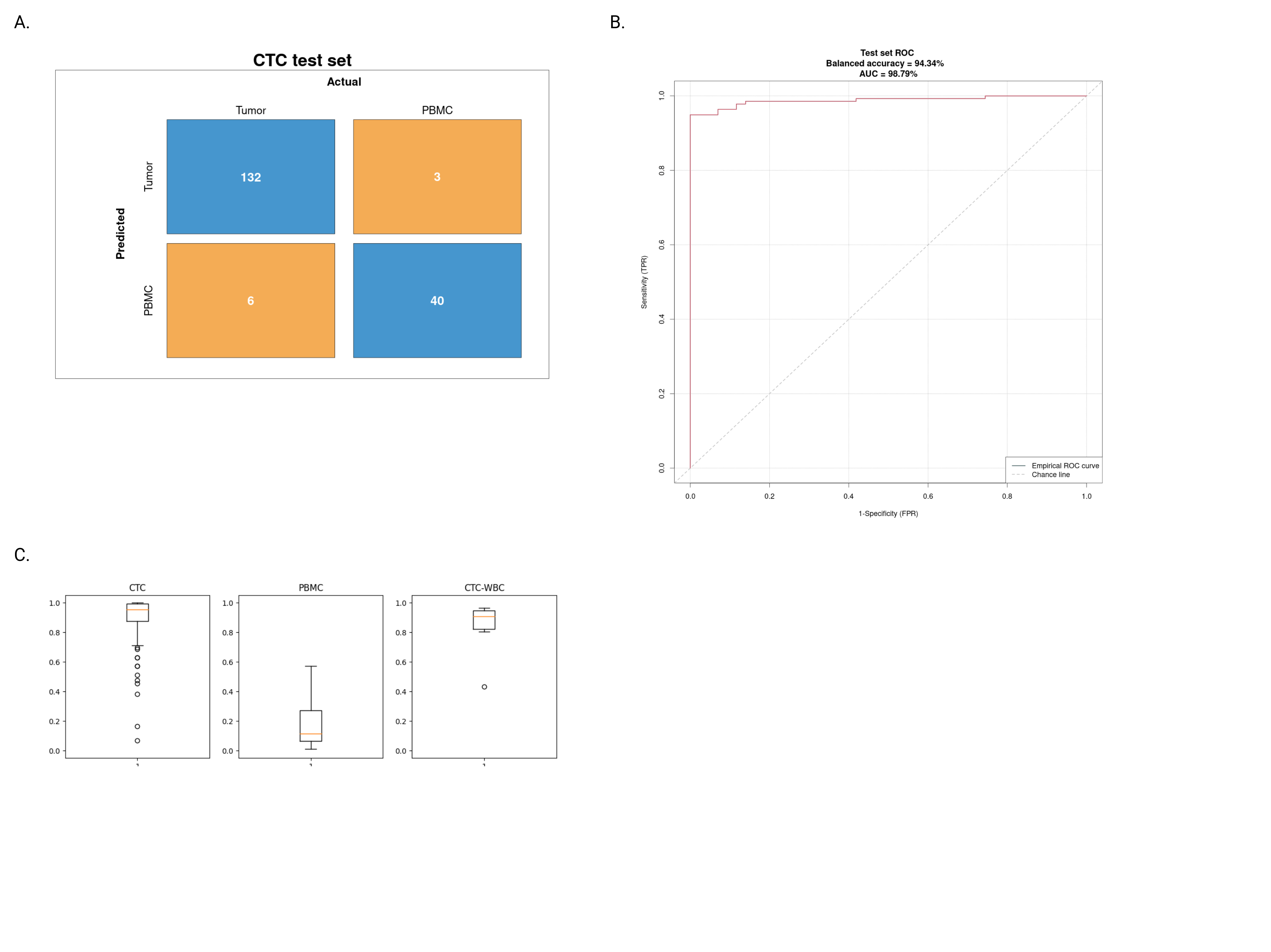

Supplement: Supplementary file 8 — Supplementary Figure 6. [file 41598_2024_61378_MOESM8_ESM.tiff]

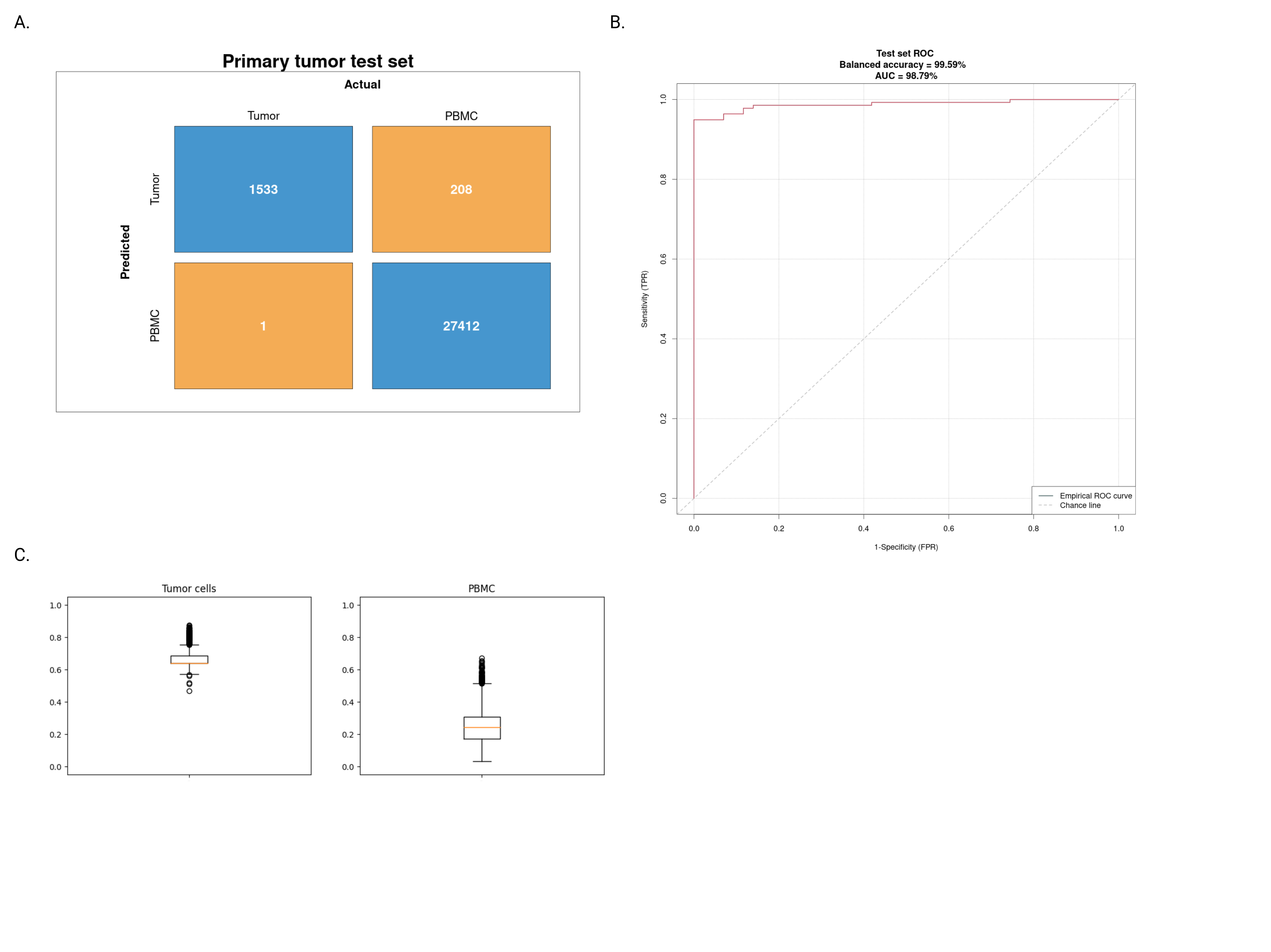

Supplement: Supplementary file 9 — Supplementary Figure 7. [file 41598_2024_61378_MOESM9_ESM.tiff]

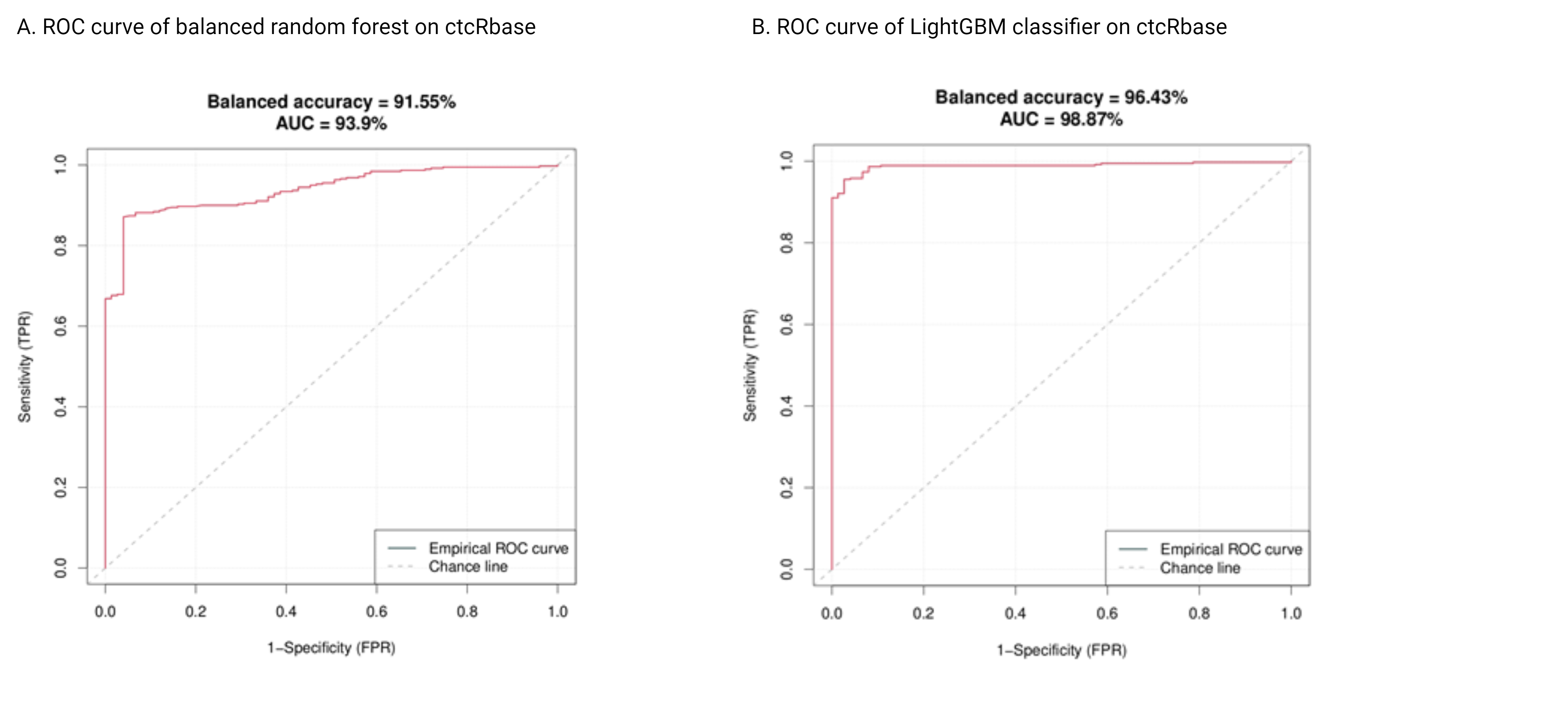

Supplement: Supplementary file 10 — Supplementary Figure 8. [file 41598_2024_61378_MOESM10_ESM.tiff]

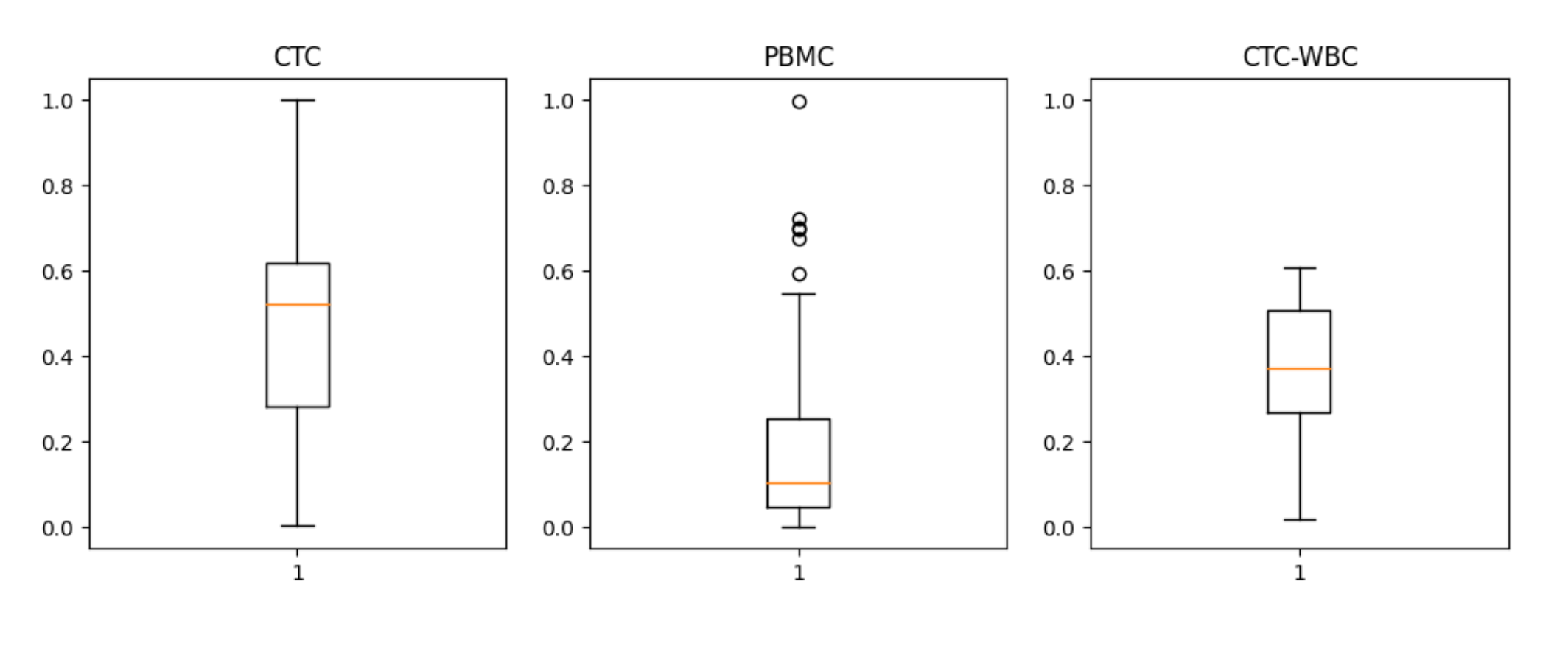

Supplement: Supplementary file 11 — Supplementary Figure 9. [file 41598_2024_61378_MOESM11_ESM.tiff]
